# Waste Fiber Powder Functionalized with Silver Nanoprism for Enhanced Raman Scattering Analysis

**DOI:** 10.1186/s11671-017-2118-5

**Published:** 2017-05-08

**Authors:** Bin Tang, Tian Zeng, Jun Liu, Ji Zhou, Yong Ye, Xungai Wang

**Affiliations:** 1 National Engineering Laboratory for Advanced Yarn and Fabric Formation and Clean Production, Wuhan Textile University, Wuhan, 430073 China; 2 Hubei Collaborative Innovation Center for Advanced Organic Chemical Materials and Key Laboratory for the Synthesis and Application of Organic Functional Molecules, Ministry of Education and College of Chemistry and Chemical Engineering, Hubei University, Wuhan, 430062 China; 3 Institute for Frontier Materials, Deakin University, Geelong, Australia

**Keywords:** Silver nanoprisms, Wool particles, Electrostatic assembly, SERS substrate, Trace analysis

## Abstract

Biomass disks based on fine powder produced from disposed wool fibers were prepared for surface-enhanced Raman scattering (SERS). The wool powders (WPs) were modified by silver nanoprisms via an assembly method and then pressed into disks using a hydraulic laboratory pellet press. Scanning electron microscopy (SEM), X-ray diffraction (XRD), and X-ray photoelectron spectroscopy (XPS) were used to characterize the WPs and disks before and after treatment with silver nanoparticles (AgNPs). The WPs retained porous structures after treatment with AgNPs. The silver nanoprisms on WPs were observed clearly and the localized surface plasmon resonance (LSPR) properties of silver nanoprisms led to blue color of wool powder (WP). The obtained WP disks with AgNPs were confirmed to enhance greatly the Raman signal of thiram. The SERS disks are low-cost and convenient to use, with high sensitivity. The characteristic SERS bands of 10^−8^ M thiram can be identified from WP disks containing silver nanoparticles.

## Background

The noble metal nanoparticles as common plasmonic materials possess a unique optical feature, i.e., localized surface plasmon resonance (LSPR) arising from interaction between electrons around nanoparticles and incident light [1–3]. The LSPR properties of noble metal nanoparticles are associated with their size, shape and surrounding environment [4, 5]. Among plasmonic materials, silver nanoparticles (AgNPs) have attracted extensive attentions, due to facile control synthesis and tenability of plasmonic features. Based on LSPR, AgNPs have been applied in many research fields such as surface enhanced spectroscopy [6, 7], biological sensing [8], and optoelectronic devices[9]. Surface-enhanced Raman scattering (SERS) has become one of the most important modern analytical techniques as it can provide abundant molecular information, with high sensitivity and non-destructive detection [10–13]. SERS has been demonstrated to be a reliable platform for trace analysis of specific molecules, including food toxins [14], environmental pollutants [15], and bioanalysis [16]. The enormous enhancement of Raman signal is originated predominantly from the electromagnetic amplification on noble metal nanostructures when the analytes are adsorbed on plasmonic metal nanoparticles [12, 17, 18]. Fabricating high SERS-active substrates with plasmonic nanoparticles is significant for development of Raman spectroscopy in practical applications.

A number of strategies have been attempted to prepare different types of SERS substrates including electrochemical or lithographical patterns, colloidal metal nanoparticle aggregates/assemblies and individual metal nanoparticles [19–23]. However, fabrication of eco-friendly and low-cost SERS substrates is still desirable for further application of SERS. Some SERS substrates have been developed through combining noble metal nanoparticles with biomaterials including cellulose and silk. Zhang et al. coated silver nanolayers on papers using physical vapor deposition (PVD) to obtain SERS test strips [24]. Moreover, SERS substrates consisting of paper and gold nanoparticles (AuNPs) were developed by Ngo et al. [25]. The papers with AuNPs were also applied for bio-diagnosis [16]. Besides, Gong et al. treated cotton swabs with AgNPs to obtain SERS substrates [26]. In addition to cellulose materials, protein materials were used to develop biomass SERS substrates. Silk film embedded with AuNPs was prepared via spin-coating mixture solution of trifluoroacetic acid (TFA) and AuNP–silk nanocomposite [27]. Subsequently, the composite film was used as substrate for SERS.

Wool as a natural protein fiber has been widely used in the textile industry because of its unique properties. A large amount of fiber waste is also produced during fiber and textile processing [28]. Converting fibers into microscaled ultra-fine particles is an alternate pathway to utilize disposed fibers. Milling has been applied to realize preparation of microstructural particles from fibers, avoiding long and costly chemical routes and use of harmful reagents [29]. Wool powders (WPs) from milling retain most of microstructure and properties of wool fiber. It has been demonstrated that converting fibers into particles increases the material surface area, resulting in significant improvements in their reactivity and absorbency, which have promoted the applications of fibrous materials in both textile and non-textile fields [30–32]. For example, WPs could be used as a sorbent to remove metal ions from solution. It was suggested that WPs have potential for application in separation and recovery of metal ions from industrial effluents and environmental waterways [31, 32]. WP as a reducing agent in-situ synthesized AuNPs and the complex of WP and AuNPs showed remarkable catalytic activity to accelerate the reduction reaction of 4-nitrophenol by sodium borohydride (NaBH_4_) [33]. Biomass fibers including wool, silk and cotton have been modified with nanoparticles to exhibit different functions such as antibacterial, UV-blocking and flame retarding features. In our previous work, anisotropic AgNPs were assembled on wool fibers to impart different colors and antibacterial properties to wool [34, 35]. The assembly of AgNPs on wool fibers was suggested to be due to the electrostatic attraction between the oppositely charged nanoparticles and fiber surface under acidic condition [34]. Compared with wool fibers, WPs have a porous structure, a large surface area and high reactivity [29], which would enhance the adsorption ability of WPs to nanoparticles. It is significant to explore the combination of WPs with functional nanoparticles and potential applications of such a combination.

Herein, an assemble method based on electrostatic interaction was developed to fabricate a novel SERS substrate from wool powder (WP), a microstructural biomass material. silver nanoprisms (AgNPrs) combined with WPs via an assembly process in aqueous solution. Circular disks consisted of plasmonic material and biomass micron particles were obtained through pressing AgNPr treated WPs. The structures and components of modified WPs were characterized using scanning electron microscopy (SEM), X-ray diffraction (XRD) and X-ray photoelectron spectroscopy (XPS). UV-vis reflection absorption spectroscopy was also employed to investigate the optical properties of disklike WP samples. Significantly, the obtained WP disks with AgNPrs were used to adsorb analyte (thiram) in ethanol and enhance of Raman signals of analytes.

## Method﻿s

### Materials

﻿AgN﻿O_3_ (>99%), trisodium citrate (≥99.0%) and sodium borohydride (>98%) were purchased from Sigma-Aldrich. Acetic acid (≥99.7%) and thiram were obtained from Sinopharm Chemical Regent Co., Ltd.. All chemicals were of analytical grade and used as received. WPs were prepared through ball milling wool fibers ac﻿cording to the procedure in an early report [29].

### Characterization

﻿Extinction spectra of AgNP solutions were recorded using a Varian Cary 3E UV/vis spectrophotometer. The UV-vis diffuse reflection absorption spectra of samples were recorded by a Varian Cary 5000 UV-VIS-NIR spectrophotometer with a diffuse reflectance accessory (DRA-2500). SEM measurements were performed with a Supra 55 VP field emission SEM. XPS measurements were carried out on a Kratos XSAM800 XPS system with Kα source and a charge neutralizer. XRD patterns were collected using a Bruker D8 Advance X-ray diffractometer with Cu Kα radiation.

### Photoinduced Synthesis of AgNPrs

AgNPrs were synthesized via a photoinduced growth process as described previously [36, 37]. First, silver seeds were prepared by dropwise addition of NaB﻿H_4_ solution (8.0 mM, 1.0 mL) to an aqueous solution of AgNO_3_ (0.1 mM, 100 mL) in the presence of trisodium citrate (1.0 mM) under vigorous stirring. The yell﻿ow silver seed solutions were then placed under a sodium lamp (NAV-T 70 model from Osram China Lighting Co., Ltd.). Eventually, blue AgNPr solutions were obtained through conversion of the yellow silver seed solution during the irradiation of sodium lamp.

### Fabrication of SERS Disks

Firstly, the pH value of the as-synthesized AgNP solution was adjusted to 4 using acetic acid. Subsequently, 0.15 g of WP was added to 750 mL of AgNP solution with pH = 4 under stirring. The weight ratio of AgNPr solution to WP was 5000. Secondly, the AgNPr solution with WPs was placed in a water bath and shaken for 1 h at 50 °C. The solution was placed at room temperature for 12 h to precipitate the blue WPs. And then the supernatant was poured out. The precipitate containing WPs was centrifuged at 6000 rpm for 6 min followed by 3 min of centrifugation at 10,000 rpm (Eppendorf, Centrifuge 5430). Finally, the blue WPs were dried using an Ecospin 3180C speed vacuum concentrator (BioTron Inc.). The dried blue WPs were ground to fine particles. Subsequently, 0.0130 g of WP was put in the pellet die and pressed at 28 MPa with a manual hydraulic press equipment (YP-2, Shanghai Shanyue Science instrument Co., Ltd.). Pure and treated WPs were pressed to circular disks through the compression process.

### SERS Measurement of Thiram

The as-prepared WP disks without and with AgNPrs were immersed into 10 mL of thiram ethanol solution with different concentrations (10^−8^ M ~ 10^−4^ M) for 12 h. After that, the disks were taken out from solution and dried under ambient condition. SERS analysis was performed on a Renishaw inVia Raman microscope system (Renishaw plc, Wotton-under-Edge, UK). A 50×/N.A. 0.75 objective and a 785-nm near-IR diode laser excitation source (500 mW, 0.5%) were used in all measurements. The spectra within a Raman shift window between 200 and 1800 cm^−1^ were recorded using a mounted CCD camera with integration time of 10 s by single scan.﻿

## Results and Discussion

### Assembly of Silver Nanoprisms on Wool Powders

**Fig. 1:**
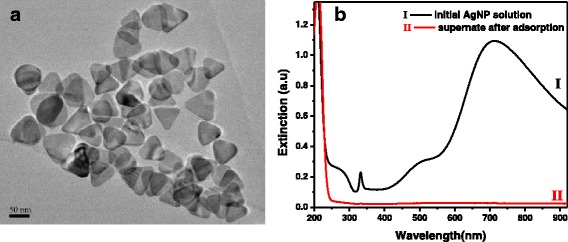
**a** TEM image of AgNPs synthesized by photoinduction. **b** UV-vis extinction spectra of silver AgNP solution before and after assembly on WPs

**Fig. 2:**
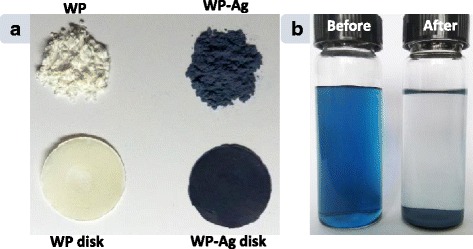
**a** Photograph of pure wool powder (WP), wool powder with AgNPrs (WP-Ag), pure wool powder disk (WP disk), and disk from wool powder treated with AgNPrs (WP-Ag disk). **b** Photograph the AgNPr solutions before and after assembly of AgNPrs on WP. Weight ratio of AgNPr solution to WP was 5000

The AgNPs prepared by light irradiation of sodium lamp were triangular nanoplates (i.e. nanoprisms) as shown in TEM image (Fig. 1a). The silver nanoprisms (AgNPrs) had blue color because of their plasmonic features. Figure 1b shows the extinction spectrum of AgNP solution (Curve I). There are three LSPR bands centered at 331 (weak), 507 (medium), and 710 nm (strong) in the extinction spectrum, assigned to the out-of-plane quadrupole, in-plane quadrupole and in-plane dipole plasmon resonance modes of triangular silver nanoplate, respectively [38, 39]. Owing to the strong plasmonic bands at long wavelength, high SERS activity from AgNPs can be obtained under the excitation laser in the near-IR region [40, 41]. WPs were prepared through ball milling of waste wool fibers. It is well known that the wool as a protein fiber has of abundant amino and carboxyl groups. Conversion from wool fibers to small particles would expose many active groups of wool. The surface charges of wool are related to the pH value [42]. It was reported previously by us that the AgNPs with different LSPR properties could be assembled on wool fibers to achieve the functionalization of wool through electrostatic interaction [34]. Similarly, the as-synthesized AgNPrs in the present study carry negative charges which can bond to a surface with positive charges. WPs carry positive charges when the pH value of reaction solution was approximately 4, due to protonation of inherent amino groups [34, 42]. Therefore, the AgNPrs can be assembled on the WPs through electrostatic interaction force between them. The white WPs changed to blue after adsorption of AgNPrs (Fig. 2a). As can be seen, the treated WPs inherited the blue color from the AgNPr solution, which indicates that the self-assembly process does not change the morphologies of AgNPrs. Moreover, the reaction solution was colorless after the adsorption of AgNPs on WPs, implying few or no AgNPs were left in the solution (Fig. 2b). There was no observable absorption band in the extinction spectrum of reaction solution after the AgNP adsorption by WPs was finished (Curve II in Fig. 1b), which further indicates that almost all AgNPrs have been adsorbed by WPs. Additionally, when the weight ratio of AgNPr solution to WP was increased to 10,000:1, WPs likewise adsorbed all AgNPs (data not shown). It has been demonstrated that WPs effectively adsorbed metal ions as a sorbent in the previous work [31]. In this research, the results reveal that WP has excellent adsorption ability to AgNPs, which is attributed to plentiful exposed internal groups and large surface area of microstructural WPs. Pure and modified WPs were pressed to circular disks using the hydraulic press equipment (Fig. 1a).

### Characterization and Analysis of SERS Disks

**Fig. 3:**
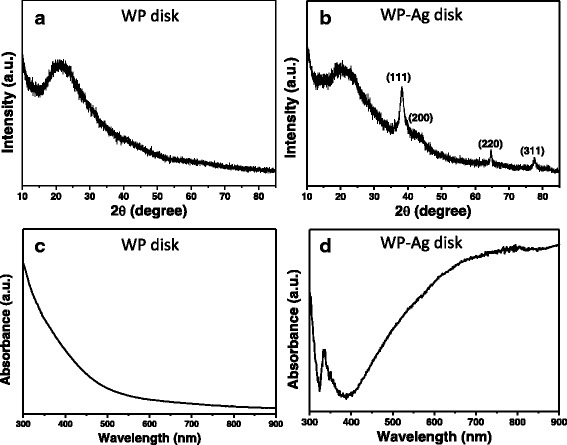
**a**, **b** XRD patterns and **c**, **d** UV-vis reflection absorption spectra of disk from pure wool powder (WP disk) and disk from wool powder treated with AgNPrs (WP-Ag disk)

Figure 3a, b displays the XRD patterns of circular WP disks before and after treatment with AgNPrs. A 2θ diffraction peak appeared around 21° in the XRD pattern of untreated WP disk (Fig. 3a), which is a characteristic XRD peak of wool [43, 44]. Compared with XRD pattern of untreated WP disk, three obvious new XRD diffraction peaks appeared in the XRD pattern of WP-Ag disk (Fig. 3b), which are assigned to (111), (200), (220), and (311) crystal planes of silver [45]. These results suggest that the AgNPrs were assembled on the WPs. In addition, the XRD peak around 21° did not change visibly after assembly of AgNPrs, which demonstrates that crystal structures of WPs remained unchanged during treatment with AgNPrs. To investigate the optical properties of WPs, UV-vis reflection absorption spectroscopy was used to measure spectral changes before and after treatment with AgNPrs. The disk from pure wool powder (WP disk) did not show observable absorption bands in the UV-vis region (Fig. 3c), which can be inferred from its white color. The reflection absorption spectrum of WP-Ag disk in Fig. 3d displays a sharp absorption peak centered at 335 nm and a broad absorption band located at more than 730 nm, which correspond to out-of-plane quadrupole (at 331 nm in solution) and in-plane dipole (at 711 nm in solution) plasmon resonance modes of AgNPrs, respectively. The AgNPs in WP-Ag disk still exhibited the typical LSPR spectrum of triangular silver nanoplates [34, 38, 39]. As can be observed, compared with the LSPR bands of AgNPr solution, the reflection absorption bands of WP-Ag disk show observable red-shift, which is because of the changes of surroundings around nanoparticles [34]. The reflectance spectroscopic results imply that assembly process of AgNPrs on WPs did not change the morphologies of nanoparticles.

**Fig. 4:**
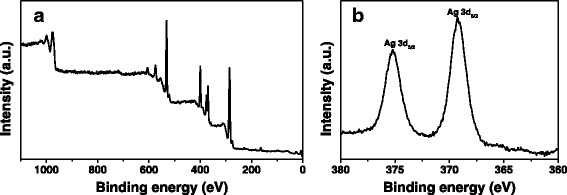
XPS spectra of disk from wool powders treated with AgNPs (WP-Ag disk): (**a**) survey; (**b**) Ag 3d﻿

We further used XPS to analyze the WP-Ag disk (Fig. 4). The normal components of the wool appeared as peaks of (O 1 s), (N 1 s), (C 1 s), and (S 2p) in the XPS spectrum (Fig. 4a). The peaks of (Ag 3d) presented in the XPS data of WP-Ag disk (Fig. 4b), revealing the existence of AgNPs on the modified WP. The XPS results prove that the AgNPrs were successfully combined with WPs.

**Fig. 5:**
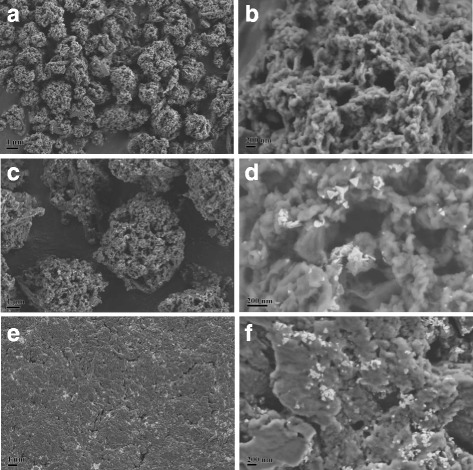
SEM images of different samples: **a**, **b** pure WPs, **c**, **d** WPs treated with AgNPs (WP-Ag), **e**, **f** disks from wool powder treated with AgNPs (WP-Ag disk)

To investigate the morphologies of the WPs during assembly of AgNPs, SEM was employed to observe the microstructures of samples. Figure 5a, b shows the SEM images of pure WPs before treatment with AgNPrs. All the particles appear separated in vision field and the size of WPs was measured to be 5.4 ± 0.8 μm (Fig. 5a). It can be seen from enlarged SEM image (Fig. 5b) that the micron particles have porous structures which can easily adsorb other substances in surroundings [29, 46]. Very recently, TEM characterization of ultrathin sections of wool powder prepared by an ultramicrotomy technique also demonstrated porous feature of wool powder [33]. Additionally, the large specific surface area of wool powder was confirmed by Brunauer–Emmett–Teller (BET) method in the previous research [29]. The large surface area and porous feature enhance the reactivity of wool microstructural particles [47]. Besides, Fig. 5c, d presents the SEM images of WP-Ag. The shape and size of WPs did not change visibly after treatment with AgNPrs (Fig. 5c), implying that the assembly process of AgNPrs did not alter the morphologies of WPs. Moreover, it can be seen that the AgNPrs were assembled on WPs (Fig. 5c, d). The SEM images show clearly that nearly all AgNPs were triangular nanoplates, which indicates that the morphologies of AgNPrs were unchanged during the assembly process. These SEM results demonstrate that the AgNPrs were bound to WPs without morphological changes. Moreover, the assembly of AgNPrs did not give rise to changes in the porous structure of WPs (Fig. 5c, d).

Figure 5e, f displays the surface SEM images of WP-Ag disk. The surface of disk was found to be smooth (Fig. 5e), due to the high pressure used to prepare the disks. Whereas, the porous structures can still be seen on the disks (Fig. 5f), which implies that the disks were porous with high reactivity. AgNPrs were observed clearly on WPs in disks, suggesting that the pressing process did not bring about the morphological variation of AgNPrs on WPs. The assembly of AgNPs led to the blue color of wool powder disks, which is consistent with conclusion from spectral characterization (Fig. 3d).

### SERS Activity of WP-Ag Disks

Thiram (bis-(dimethyldithiocarbamoyl) disulfide) is a pesticide, widely used to protect crops from downy mildew, blight, anthracnose and cereal smut [48]. The thiram residues on the surface of foods not only pollute the environment, but also are irritant for the eyes, skin and respiratory tract [49, 50]. It is necessary to evaluate the risk of thiram residues through detection of trace quantities of thiram. Some methods have been used for the determination of thiram, such as chromatography [51], enzyme linked immunosorbent assay [52] and chemiluminescence analysis [53]. SERS is one effective method to determine the trace residue of thiram [48, 54, 55, 56]. LSPR properties of AgNPs influence remarkably the SERS activity, which has be widely investigated by researchers [40, 41]. The higher SERS enhancement would be obtained when the LSPR band of SERS substrate overlaps more with the wavelength of excitation laser during Raman testing. In the present study, the obtained SERS disk (WP-Ag disk) displays a broad LSPR band at a long wavelength (more than 730 nm as shown in Fig. 3d), due to assembly of silver nanoprisms. Therefore, 785-nm laser was chosen as excitation light source for SERS test in this work.

**Fig. 6:**
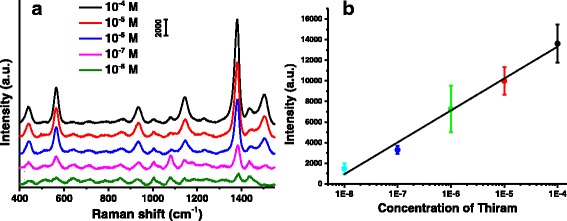
**a** SERS spectra of thiram with different concentrations obtained from WP-Ag disks. The shown SERS spectra are average results from ten spectra recorded. Before averaging, the raw spectra were background-corrected. **b** Plot of the intensities of SERS signals at 1381 cm^−1^ as a function of the concentrations of thiram in ethanol. Each *point* in the plot represents the average value of ten random measurements on WP-Ag disks and *error bars* indicate the standard deviation for each set of measurements. The *line* is the linear fitting curve based on corresponding average values

The porous structures, large surface and high reactivity of WPs would favor adsorption of analytes. Moreover, different from dispersive particles, the pressed WP-Ag disks can be easily taken out from a solution system. The disks consisted of the assembly of WPs and AgNPrs were used for SERS substrates to detect trace thiram in ethanol solution. Figure 6a shows the SERS spectra of thiram with different concentrations (10^−8^ M ~ 10^−4^ M) obtained from WP-Ag disks. Table 1 displays the details of the assignments of the different SERS bands of thiram [55]. Among the SERS bands, the band at 1381 cm^−1^ is the strongest, which is the CN stretching mode and symmetric CH_3_ deformation mode. The band at 1437 cm^−1^ is assigned to the antisymmetric stretching mode of CH_3_. The bands at 1505 cm^−1^ and 1144 cm^−1^ are associated with the CN stretching vibrations and rocking CH_3_ mode. The peak at 935 cm^−1^ is due to the C = S and CH_3_N stretching vibrations. The bands at 568 cm^−1^ is attributed to the SS stretching mode. The band at 439 cm^−1^ is ascribed to the CH_3_NC deformation and C = S stretching mode [57]. These characteristic SERS bands increased as the concentration of thiram increased. It can be seen that the band at 1381 cm^−1^ can be identified even when the concentration of thiram is 10^−8^ M, which indicates that the WP-Ag disks have strong SERS activity. Figure 6b depicts the plot of the intensity of band at 1381 cm^−1^ as a function of concentration of thiram. It is found that there is a good linear relationship between SERS intensities (at 1381 cm^−1^) and the logarithm of thiram concentration.

**Table 1:** Detail of assignment of SERS spectra of thiram

Wavenumber (cm^−1^)	Assignment
439	δ (CH_3_NC), ν (C = S)
568	ν (SS)
935	ν (CH_3_N), ν (C = S)
1144	ρ (CH_3_), ν (CN)
1381	δ (CH_3_), ν (CN)
1437	δ as(CH_3_)
1505	ρ (CH_3_), ν (CN)

**Fig. 7:**
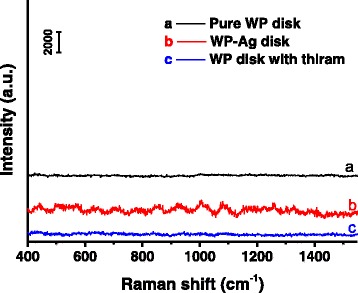
Raman spectra of (Curve a) pure WP disk, (Curve b) WP-Ag disk and (Curve c) pure WP disk after being immersed in 10^−4^ M of thiram solution for 12 h (WP disk with thiram)

WPs as protein materials may disturb the SERS testing of thiram. Figure 7 displays the Raman spectrum of pure WP disk (Curve a) under the same testing conditions as collection of SERS. No evident bands were observed in the Raman spectrum of pure WP disk. In addition, the WP-Ag disk without thiram was also tested using Raman spectroscopy (Curve b in Fig. 7). Although some weak bands occurred due to enhancement effect from AgNPrs, no characteristic Raman bands of thiram appeared. The results suggest that Raman signal from biomass substrates should not affect notably the SERS spectra of analytes. In addition, the pure WP disk after immersing in 10^−4^ M of thiram solution for 12 h was tested by Raman spectroscopy. No observable bands were seen in the Raman spectrum (Curve c in Fig. 7), implying the AgNPrs play a pivotal role for the SERS enhancement.

## Conclusions

A novel surface-enhanced Raman scattering (SERS) substrate was fabricated through assembly of silver nanoprisms (AgNPrs) on wool powders (WPs) followed by hydraulic pellet press treatment. XRD and XPS demonstrated that AgNPrs have been bound to WPs. It was confirmed that the assembly process did not change visibly the morphologies of WPs and AgNPrs. Moreover, the WPs have great adsorption ability for AgNPrs. The properties of wool powder were retained in the disks with AgNPs (WP-Ag disks), which favors adsorption of analytes. The LSPR features of AgNPrs were transferred to wool powder after assembly process. The WP-Ag disks show strong enhancement activity for Raman signal of thiram. With the inherent ability to separate and concentrate AgNPrs and analytes, WPs are not only porous supports but also integral analytical platforms containing pretreatment and SERS detection, which may have potential applications in field studies and point-of-care testing combining a portable Raman spectrometer. The complex of microstructural particles from natural fibers and functional nanoparticles would expand the application scope of biomass materials.
